# An estimation of distribution algorithm with clustering for scenario-based robust financial optimization

**DOI:** 10.1007/s40747-021-00640-2

**Published:** 2022-03-05

**Authors:** Wen Shi, Xiao-Min Hu, Wei-Neng Chen

**Affiliations:** 1 School of Computer Science and Engineering, South China University of Technology, Guangzhou, China; 2 School of Computers, Guangdong University of Technology, Guangzhou, China; 3 Pazhou Lab, Guangzhou, 510330 China

**Keywords:** Multi-objective optimization, Uncertainty handling, Estimation of distribution algorithm, Financial investment

## Abstract

One important problem in financial optimization is to search for robust investment plans that can maximize return while minimizing risk. The market environment, namely the scenario of the problem in optimization, always affects the return and risk of an investment plan. Those financial optimization problems that the performance of the investment plans largely depends on the scenarios are defined as scenario-based optimization problems. This kind of uncertainty is called scenario-based uncertainty. The consideration of scenario-based uncertainty in multi-objective optimization problem is a largely under explored domain. In this paper, a nondominated sorting estimation of distribution algorithm with clustering (NSEDA-C) is proposed to deal with scenario-based robust financial problems. A robust group insurance portfolio problem is taken as an instance to study the features of scenario-based robust financial problems. A simplified simulation method is applied to measure the return while an estimation model is devised to measure the risk. Applications of the NSEDA-C on the group insurance portfolio problem for real-world insurance products have validated the effectiveness of the proposed algorithm.

## Introduction

Uncertainty is ubiquitous in financial optimization problems. The uncertainties in financial optimization problems lead to uncertain returns of investments. Therefore, in the issue of financial investment, investors always aim at searching for robust investment plans that can not only gain a considerable return but also avoid excessive risk in uncertain environments. That is to say, many financial optimization problems can be formulated as robust optimization problems with two objectives, return maximization and risk minimization [1–4]. The main challenge in robust financial optimization problems is to deal with the uncertainties in investment properly.

In general, there are four kinds of popular ways to deal with uncertainties in multi-objective problems (MOPs). First, for those problems that the fitness functions are derivable and the uncertainties can be easily expressed by mathematical formulas, the traditional mathematical derivation method is an important method to handle uncertainties [1, 2]. Second, traditional Monte Carlo (MC) simulation method is popular to deal with complex uncertainties which are difficult to express by mathematical formulas [3–7]. According to the law of large numbers, the frequency of a random event is close to its probability under the same environment and a large number of repeated experiments. Therefore, the results of a large number of MC simulations can be employed to approximate the objectives of the problem. The accuracy of the estimation increases with the number of simulation according to the law of large numbers. Third, for those problems that the uncertainties obey certain probability distribution such as the noise with normal distribution, one popular approach is to estimate the expectation value of the solution by the average value of each parameter [8]. Last, surrogate models such as radial-basis-function network [9], back propagation neural network [10] and general regression neural network [11] are also widely applied to deal with uncertainties in MOPs especially to approximate the risk due to their good fitting ability [12, 13].

Despite the above methods, a special scenario-based feature in financial optimization has been noticed [14]. In most of financial optimization problems, the influence of the scenario on the result is greater than the quality of the solution. In other words, the return of a good investment plan in the negative market (i.e., most of the products are not profitable) may be worse than the return of a bad investment plan in the positive market (i.e., most of the products can gain considerable profits). For instance, with the outbreak of COVID-19, the financial market is greatly impacted. Even though the best investment plan under the COVID-19 circumstance cannot get the same return as a bad investment plan in a normal circumstance [15]. Another example is that in severe drought years, even with the optimal planting scheme, the harvests of cereals are not as well as an unsuitable planting scheme in a harvest year [16]. These kinds of scenario-based uncertainties commonly appear in various robust financial optimization problems whose objective is minimizing risk while maximizing return. The special characteristics of scenario-based uncertainties make the existing approaches inefficient.

First, due to the complexity of scenario-based uncertainty, the problem is difficult to express by a precise mathematical model. Therefore, traditional mathematical derivation methods are difficult to apply in this kind of uncertainty problem. Second, traditional MC simulation method is not effective for the scenario-based robust optimization problem since huge amounts of simulations are needed to stabilize the expectation value in financial problems, which are time-consuming. Third, for the scenario-based robust optimization problem considered in this paper, the uncertainties are influenced by a lot of factors and do not obey certain distribution. Therefore, the estimation approach is not applicable to this problem. Last, according to our analyses, the surrogate models are ineffective to approximate the risk considered in this paper due to the scenario-based characteristics of the uncertainty. The methods for solving uncertainty in MOPs and their drawbacks are concluded in Table 1.

**Table 1:** Methods for solving uncertainty in MOPs and their drawbacks

Methods	Scope of application	Drawbacks
Mathematical derivation method	Problems that the fitness value are derivable and the uncertainties can be expressed by mathematical formulas	The scenario-based uncertain optimization problems are difficult to express by a precise mathematical model
Traditional MC simulation method	Problems which are difficult to express by mathematical formulas	Huge amounts of simulations are needed to stabilize the expectation value of the problems, which are time-consuming
Estimation with average parameter	Problems that the uncertainties obey certain probability distribution such as the noise with normal distribution	The uncertainties in the problems are influenced by a lot of factors and do not obey certain distribution
Surrogate model	Approximation problems of the risk	Ineffective due to the scenario-based characteristics of the uncertainty

To cope with this special kind of scenario-based robust optimization problem, a nondominated sorting estimation of distribution algorithm with clustering (NSEDA-C) is proposed in this paper. An insurance portfolio problem [17] is studied as an instance of the multi-objective robust financial optimization problem. The nondominated sorting (NS) approach [18], which has been widely applied to address MOP is applied to cooperate with the estimation of distribution algorithm (EDA) [19]. EDA is a stochastic optimization algorithm with great ability to deal with uncertain optimization problems due to the compatibility of its inherent stochastic and the uncertainties in the optimization problem [20]. Moreover, a clustering method [21] is applied to find more peaks during the evolution process. The population in NSEDA is divided into several clusters according to Euclidean distance [22]. The distribution models are constructed in each cluster independently. In view of the scenario-based feature in robust financial optimization problems, this paper introduces two novel strategies to address the estimation of return and risk based on feature analysis of the problem.

On the one hand, to coordinate with the scenario-based characteristic of the uncertainty, a simplified simulation strategy is proposed to reduce the number of simulations when estimating the return. In this kind of uncertain optimization problem, although there are huge fluctuations in the market, a good portfolio plan can withstand the fluctuations and outperforms the poor portfolio plans in the same scenario for most of the time. Therefore, in the evolution process, the quality of the solutions can be distinguished by only one simulation, which can save a huge amount of computational resources.

On the other hand, a data-driven heuristic estimation model is devised to measure the risk of the problem. Although the calculation of the return can be simplified to one simulation due to its scenario-based characteristics, the risk of the portfolio plan is incalculable by only one simulation. In the proposed NSEDA-C, since only an approximative rank of the variance is needed to find Pareto-optimal solutions, a problem-heuristic estimation model is constructed based on the historical data to approximate the rank of the variance. The accuracy of the estimation model is measured by the Spearman rank correlation coefficient [23]. A high Spearman rank correlation coefficient indicates that it is effective to apply the proposed estimation model as a substitute for the variance when searching for the Pareto-optimal solutions.

The remaining part of the paper proceeds as follows: background information about MOPs and robust financial optimization problems is given in “2”. “5” gives a specific example of the discussed scenario-based robust optimization problems and analyzes the problem thoroughly based on the given example. “Nondominated sorting estimation of distribution algorithm” elaborates the proposed NSEDA-C. Experiments are conducted in “18” where the proposed NSEDA-C is compared with other algorithms. Finally, the conclusion of the whole article is summarized in “26”.

## Backgrounds

Since the problem considered in this paper is essentially a MOP, background information about MOPs is given in this section for those who are not familiar with MOPs. After that, we give a further description of the considered robust financial optimization problems and review some popular approaches to deal with robust financial optimization problems.

### Multi-objective problems

MOPs [24–26] have been widely applied in a large number of fields in real life, such as transportation problem [27, 28], recommendation system [29], shortest path problem [30], and analog design automation [31]. In general, a MOP with *m* decision variables and *n* objectives are defined as follows [32]:

1 $$\begin{gathered} {\text{max }}f(x) = (f_{1} (x),f_{2} (x),...f_{n} (x)) \hfill \\ {\text{subject to }}x = (x_{1} ,x_{2} ,...x_{m} ) \in \Omega , \hfill \\ \end{gathered}$$

where Ω ⊆ *R*^*m*^ is the search space. Here, the objectives are contradictious, which means that no solution can reach the optimal value for all the objectives simultaneously. For two solutions, *s*_1_ is said to dominate *s*_2_ if and only if

2 $$\begin{gathered} \, \forall i \in \{ 1,2,...n\} : \, f_{i} (s_{1} ) \ge f_{i} (s_{2} ) \hfill \\ \wedge \, \exists j \in \{ 1,2,...n\} : \, f_{i} (s_{1} ) > f_{i} (s_{2} ). \hfill \\ \end{gathered}$$

If a solution *s* is not dominated by any other solutions in a set Ω′, *s* is said to be a nondominated solution in Ω′. *s* is Pareto-optimal solution if and only if *s* is nondominated in the whole search space Ω. The set of objective values corresponding to a set of Pareto-optimal solutions is called Pareto-optimal front.

Efforts have been made to find the Pareto-optimal solutions and the Pareto-optimal front for MOPs by evolutionary computation. Among these algorithms, the most popular two works are nondominated sorting genetic algorithm II (NSGA-II) [18] and MOEA based on decomposition (MOEA/D) [33]. NSGA-II propose a fast nondominated sorting approach which improves the computational complexity of Pareto dominated sorting from *O*(*NP*^3^) to *O*(*NP*^2^), where *NP* is the population size. MOEA/D has addressed the issue that NSGA-II is inefficient when dealing with high dimensional MOPs. In MOEA/D, a MOP is decomposed into several scalar optimization subproblems by uniformly distributed weight vectors. For each newly generated solution, solutions near the subproblem are replaced by an aggregate function.

Treatments of noise are the most important problems in uncertain MOPs. In noisy MOPs, the evaluation of the fitness function is disturbed by noise such as loss during image processing [34], transmission noise [35] or line crosstalk noise [36]. Noisy MOPs can be described as the following equation:

3 $$\begin{gathered} \max \hat{f}(x) = \left( {f^{\prime}_{{1}} (x),f^{\prime}_{{2}} (x),...f^{\prime}_{n} (x)} \right) \hfill \\ {\text{where }}f^{\prime}_{i} (x) = f_{i} (x) + N(0,\sigma_{i}^{2} ){\text{ or }}f^{\prime}_{i} (x) = f_{i} (x) \times N(0,\sigma_{i}^{2} ). \hfill \\ \end{gathered}$$

Here, *f*_*i*_′(*x*) = *f*_*i*_(*x*) + *N*(0, *σ*_*i*_^2^) stands for additive noise while *f*_*i*_′(*x*) = *f*_*i*_(*x*) × *N*(0, *σ*_*i*_^2^) stands for multiplicative noise. Generally, the noise in the function is assumed to be normally distributed with mean value 0 and standard deviation *σ*_*i*_.

The most common method to deal with noisy MOPs is to approximate the fitness value with MC simulation. For example, Babbar et al*.* [7] proposed a modified NSGA-II that applied a clustering method to the ranking scheme in NSGA-II to select and evolve nondominated solutions over the objective space. The performances of the algorithms in noisy environments are improved a lot by the proposed method. Their work is then followed by Boonma and Suzuki [6] with the introduced of a noise-aware dominance operator called α-dominance operator. The proposed operator identified the dominance relationship between two candidate solutions by statistically processing their objective value samples. After that, Rakshit et al*.* [5] modified the traditional differential evolution for multi-objective (DEMO) [37] in the presence of noise by three strategies: adaptive sample size selection, consideration of determining statistical expectation as the measurement and consideration of slightly worse trial solutions. Then, they improved their work by employing determining defuzzified centroid value of the samples as the measurement of fitness value and proposed an extending DEMO for optimization in the presence of noise [38]. Especially, Wang et al*.* [8] paid attention to the regularity property of the Pareto set in MOPs and embedded the regularity model in NSGA-II to cope with noises.

### Robust financial optimization problems

Robust financial optimization problems are a special kind of MOP that usually consist of two objectives. The first objective is the return on investment, which needs to be maximized. The second objective is the risk of investment, which needs to be minimized. In general, robust financial optimization problem can be formulated as

4 $$\begin{gathered} {\text{max }}f(x) = (f_{1} (x),f_{2} (x)){\text{ where}} \hfill \\ f_{1} (x) = return{\text{ and }}f_{2} (x) = - risk. \hfill \\ \end{gathered}$$

The most traditional method is applying mathematical derivation strategies to solve robust financial optimization problems. For example, Patnaik and Tiwari [1] applied Value-at-Risk approach to measure market risk which helps to find out robust solutions that can maximize the return while minimize market risk. Duan [2] applied convex vector optimization to deal with multi-objective portfolio problem and search for robust solutions with return maximization and risk minimization.

Another popular approach is to simulate the investment process with MC simulation. For example, Mukerjee et al. [3] applied a nondominated sorting genetic algorithm (NSGA-II) cooperates with MC simulation to handle the robust risk-return trade-off problem which searches for robust solutions that can make a balance between the risk and the return in bank loan management. Lin et al. [4] proposed a mean–variance model with simulation to search for robust solution that can rebalance the transaction costs and minimum transaction lots.

However, few of existing literature has paid attention to the scenario-based uncertainties in robust financial optimization problems and devised algorithms based on this kind of uncertainties. Those existing algorithms are not effective for the scenario-based robust financial optimization problems due to the hindrance of special uncertainties in the problem. Therefore, this paper focuses on the features of scenario-based robust financial optimization problems and designs a specific optimization algorithm according to this special feature.

## Scenario-based robust optimization problem

In this section, a data-driven group insurance portfolio problem is introduced as an instance of scenario-based robust optimization problems. Analyses are made based on the group insurance portfolio problem to study the feature of scenario-based robust optimization problems.

### Group insurance portfolio problem

The study of data-driven insurance portfolio problem first started from [17] as an single-objective deterministic problem where only the maximization of the return of a single insured is considered as the objective of the problem and an approximated expectation reward is applied as the objective function. After that, an extension is made in [14, 39] which considers the optimization problem of a whole group and taken the mean value of uncertain simulation result as the objective function. Now, we further expand this problem to a two-objective robust optimization problem considering both the maximization of the return and the minimization of the risk of a whole group as the objectives.

Inspired by the measurement of risk from Markowitz’s work [40], the objective function is formulated as

5 $$\begin{gathered} {\text{max }}f({\varvec{X}},{\varvec{\beta}}) = (f_{1} ({\varvec{X}},{\varvec{\beta}}),f_{2} ({\varvec{X}},{\varvec{\beta}})) \hfill \\ {\text{where }}f_{1} ({\varvec{X}},{\varvec{\beta}}) = {\text{mean }}S({\varvec{X}},{\varvec{\beta}}) \hfill \\ \, f_{2} ({\varvec{X}},{\varvec{\beta}}) = - {\text{std }}S({\varvec{X}},{\varvec{\beta}}). \hfill \\ \end{gathered}$$

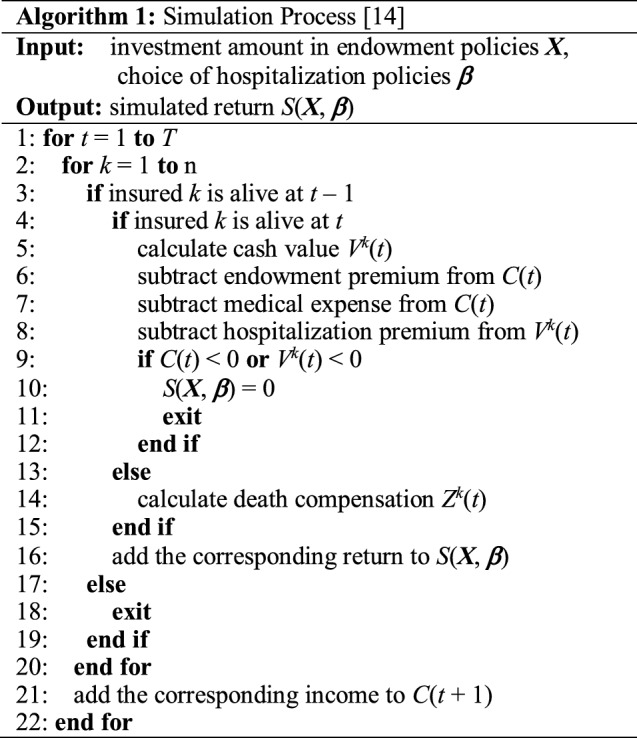


where ***X*** denotes the investment amount in endowment policies and ***β*** denotes the choice of hospitalization policies. *f*_1_ is the return of the portfolio plan which is calculated by the mean value of a huge number (ten thousand in this paper) of simulated return *S*(***X***, ***β***). *f*_2_ is the risk of the portfolio plan which is calculated by the sample standard deviation of a huge number (ten thousand in this paper) of simulated return *S*(***X***, ***β***). A brief pseudocode of the simulation process is given in Algorithm 1, which has been elaborated in [14].

### Feature analysis of group insurance portfolio problem

Analyses are made on group insurance portfolio problems in the following four situations to study the features of scenario-based robust optimization problems:

1. *t*_0_ = 30, 45, 50; *T* = 20;
2. *t*_0_ = 30, 45, 50; *T* = 30;
3. *t*_0_ = 30, 30, 35; *T* = 20;
4. *t*_0_ = 30, 30, 35; *T* = 30.

Here, *t*_0_ denotes the initial ages of different insureds in the group and *T* denotes the time duration of the investment considered in the problem.

#### Distribution of the results

First, we explore the distribution of the two objectives in the group insurance portfolio problem to verify that this problem is an optimizable multi-objective problem. On the one hand, those problems with positively related objectives can be treated as single-objective problems since when one objective is optimized, the others are also optimized. On the other hand, those problems with completely contradictory objectives are not optimizable since as long as one solution performs better than another in one objective, its performance must be worse in the other objectives.

**Fig. 1:**
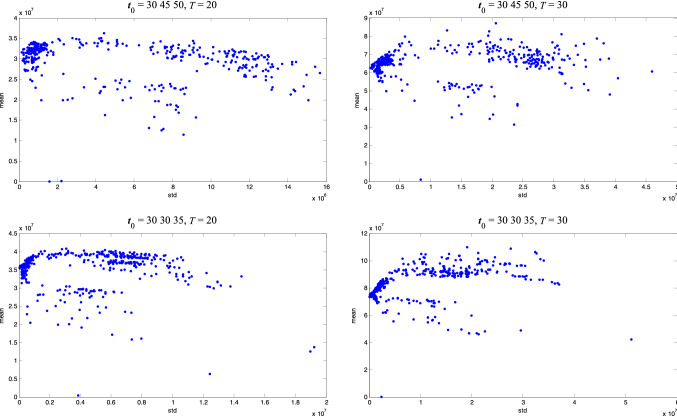
Result distribution of group insurance portfolio problem

1000 independent solutions are generated randomly and their returns and risks are estimated in 10,000 simulated scenarios. Result distribution in four situations is given in Fig. 1. It can be discovered that these two objectives are partially contradictory. Although solutions with high returns have relatively high risks in general, there are still superior solutions that not only the returns are high but also the risks are low.

To further validate the rationality of applying these two objectives to constitute a multi-objective optimization problem, the Pearson correlation coefficients between these two objectives are researched. The Pearson correlation coefficients between these two objectives in four situations are given in Table 2. Since the Pearson correlation coefficients of these two objectives are neither too high nor too low in the four situations, it is reasonable to construct a multi-objective optimization problem with these two objectives. Situation with different Pearson correlation coefficient represents multi-objective optimization problem with different difficulty. In the first and third situations, since the objectives are more consistent, it is easier to find out robust solutions than in the second and fourth situations.

**Table 2:** Pearson correlation coefficients of the objectives

*t*_0_ = 30 45 50 *T* = 20	*t*_0_ = 30 45 50 *T* = 30	*t*_0_ = 30 30 35 *T* = 20	*t*_0_ = 30 30 35 *T* = 30
0.34	− 0.29	0.12	− 0.55

#### Stability analysis of the objectives

**Fig. 2:**
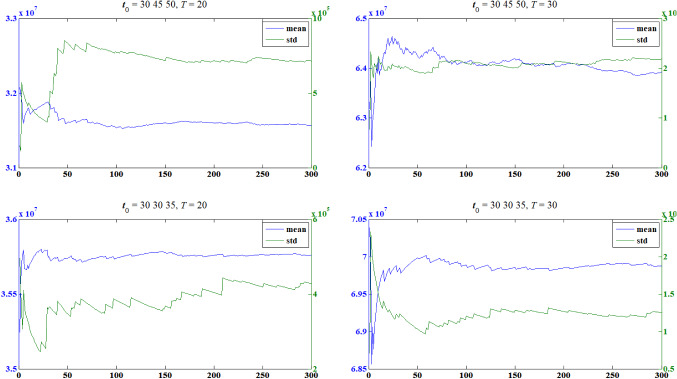
Convergence graph of the two objectives

Then, we analyze the stability of the objectives through their convergence graph. In each situation, we randomly generate one solution as a representative to carry on the study. The mean value and the standard deviation of the simulated return within 300 simulations are given in Fig. 2.

In the first situation, the mean value is stabilized after 150 simulations while the standard deviation is stabilized after 250 simulations. In the second situation, both the mean value and the standard deviation have not been stabilized within 300 simulations. In the third situation, the mean value is stabilized after 200 simulations while the standard deviation has not been stabilized within 300 simulations. In the fourth situation, both the mean value and the standard deviation have not been stabilized within 300 simulations. That is to say, more than 300 simulations are needed to gain an accurate approximation of both of the objectives in all the situations in the group insurance portfolio problem.

Since the simulation process is the most time-consuming process during optimization, it is not cost-effective to calculate the fitness value of the objectives with more than 300 simulations. Therefore, alternative approximation strategies are needed to estimate the objectives of the problem to avoid expensive computational cost.

#### Characteristic analysis of the return

Therefore, we explore the characteristics of the return by calculating the probability that the better solution outperforms the worse solution in each situation. Fifteen pairs of solutions are randomly chosen in each situation. The return of each pair of solutions is simulated in the same 10,000 scenarios. The solution with a higher mean value of the return in 10,000 scenarios is regarded as the better solution while the solution with a lower mean value is regarded as the worse solution in each pair of solutions. The ratios of the scenarios that the better solution outperforms the worse solution for each pair of solutions are given in Table 3. The mean values of the ratio in each situation are also given in Table 3.

**Table 3:** Probability that the better solution outperforms the worse solution

	Mean	P1	P2	P3	P4	P5	P6	P7	P8	P9	P10	P11	P12	P13	P15
*t*_0_ = 30 45 50 *T* = 20	0.90	0.90	0.97	0.86	0.90	0.80	0.84	0.93	0.90	0.86	0.93	0.90	0.92	0.86	1.00
*t*_0_ = 30 45 50 *T* = 30	0.89	0.85	0.82	0.96	0.97	0.84	0.93	0.93	0.90	0.86	0.93	0.86	0.90	0.91	0.86
*t*_0_ = 30 30 35 *T* = 20	0.97	0.98	1.00	0.95	0.96	0.97	0.98	0.91	1.00	0.98	0.87	0.98	1.00	0.92	0.98
*t*_0_ = 30 30 35 *T* = 30	0.97	0.99	1.00	0.99	0.94	0.97	1.00	0.97	0.96	0.93	0.95	0.97	0.91	0.99	0.97

It can be discovered in Table 3 that in the considered group insurance portfolio problem, it is very possible that a better solution will outperform a worse solution in the same simulated scenario. Due to this scenario-based characteristic of the uncertainty, a corresponding approach is designed to save computational costs, which will be elaborated later.

#### Approximation of the risk

Lastly, although the calculation of the return can be simplified due to its scenario-based characteristics, the calculation of the risk still remains a big challenge. Therefore, we search for an approximation approach of the measurement of risk. Three widely applied surrogate models are investigated including radial-basis-function (RBF) network [9], back propagation (BP) neural network [10] and general regression neural network (GRNN) [11]. Except for these three surrogate models, the accuracy of a small number of MC simulation (including 10 simulations, 20 simulations and 50 simulations) is also investigated. The approximation error is calculated by the following equation:

6 $$e = \frac{{\left| {f - f_{e} } \right|}}{{\max (f,f_{e} )}},$$

where *f* is the fitness value of risk calculated by 10,000 MC simulations and *f*_*e*_ is the approximation value of the risk. The approximation errors are given in Table 4.

**Table 4:** Approximation error of the risk

	RBF	BP	GRNN	10 *MC*	20 *MC*	50 *MC*
*t*_0_ = 30 45 50 *T* = 20	0.57	0.69	0.57	0.44	0.32	0.24
*t*_0_ = 30 45 50 *T* = 30	0.63	0.63	0.55	0.62	0.37	0.22
*t*_0_ = 30 30 35 *T* = 20	0.48	0.74	0.50	0.41	0.35	0.25
*t*_0_ = 30 30 35 *T* = 30	0.60	0.76	0.52	0.72	0.38	0.26

It can be discovered that all the surrogate models are not suitable for the approximation of the risk in group insurance portfolio problem since the approximation errors are rather large. This situation may be caused by the scenario-based characteristics of the problem. Since the uncertainty of the problem is highly related to the simulated scenario, it is hard for those surrogate models to estimate the sample standard deviation of the simulated return accurately.

As for the small number of MC simulations, the approximation accuracy of 10 simulations and 20 simulations is also poor. The accuracy of 50 simulations is relatively better, but compared with the increase in computational cost, the increase in accuracy is not enough.

Thus, another estimation approach with higher accuracy and lower computational cost is needed to estimate the risk in group insurance portfolio problem. Since the group insurance portfolio problem is constructed based on a huge number of historical data. The historical data and distribution of the payout of each endowment policy are applied to simulate the cash value or death compensation for the insured. The historical data of the mortality rate and incidence rate are applied to simulate the lifespan and health condition of the insured. Therefore, the idea of constructing a heuristic estimation model with these huge number of historical data naturally come to our mind. The detailed introduction of this data-driven heuristic estimation model will be given in the next section.

## Nondominated sorting estimation of distribution algorithm with clustering

### General framework

To solve the proposed group insurance portfolio problem with two objectives, a nondominated sorting estimation of distribution algorithm with clustering (NSEDA-C) is devised. The general framework of the proposed NSEDA-C is composed of two parts: the nondominated sorting strategy and the clustering estimation of distribution algorithm.

#### Nondominated sorting strategy

The nondominated sorting strategy applied in this paper was first proposed by Deb [18] and widely applied to solve multi-objective especially two-objective problems.
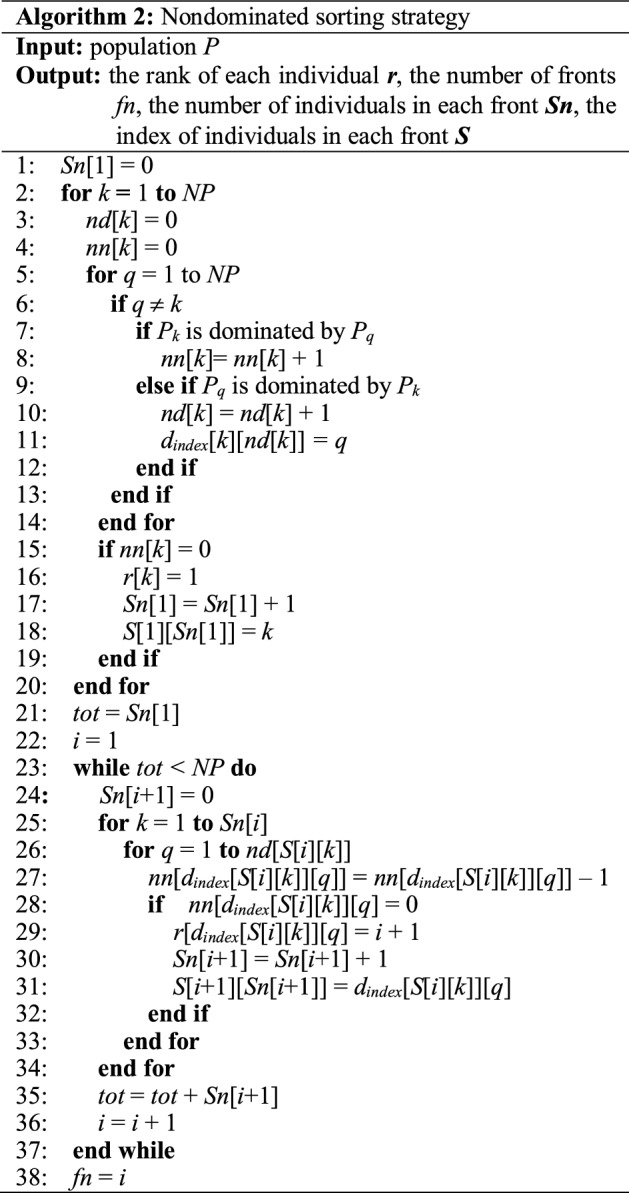


The pseudocode of nondominated sorting strategy is given in Algorithm 2. Specially, for those individuals with the same rank, diversity preservation ability is applied to distinguish the individuals. The diversity preservation ability of the individual is measured by the crowding distance for a sorted population *I* with *l* individuals:

7 $$d_{im} = \left\{ \begin{gathered} \infty {\text{ if }}i = 1{\text{ or }}i = l \hfill \\ \frac{{I_{i + 1} .m - I_{i - 1} .m{\text{ otherwise}}}}{{f_{m}^{\max } - f_{m}^{\min } }} \hfill \\ \end{gathered} \right.,$$

8 $$d_{i} { = }\sum\limits_{m = 1}^{M} {d_{im} } ,$$

where $f_{m}^{\max }$ and $f_{m}^{\min }$ are the maximum and minimum value of the *m*-th objective. *I*_*i*_.*m* denotes the *m*-th objective value of the *i*-th individual and *M* is the number of objectives in the problem. For two individuals with the same dominated rank, the one with larger crowding distance is considered better.

#### Clustering estimation of distribution algorithm

In this paper, a clustering estimation of distribution algorithm (CEDA) [14] designed for mixed variable optimization is applied as the optimizer to deal with the group insurance portfolio problem. The CEDA executes according to the following steps.

*Step 1: Initialization with constraint handling*. An initial population is generated randomly in sequence to ensure its feasibility. Each individual in the population stands for a candidate portfolio plan of the group insurance portfolio problem. Since the total investable amount of the group is fixed, the upper bound of investment amount to each endowment policy will be limited by the investment amount to other endowment policies. Therefore, the investment amount to each endowment policy is initialized one by one to ensure that the solution is feasible. Once the investment amount to an endowment policy is determined, the corresponding value will be subtracted from the remaining amount to other endowment policies according to its payment period. Moreover, to avoid the phenomenon that the investment of individuals concentrated in the first few policies, the initialization sequence is randomly disrupted for each individual.

*Step 2: Construction of probability models in each cluster*. The elite individuals in the population, which are found out by the nondominated sorting method and crowding distance are applied to construct the probability model for the next generation. After *NP*_*best*_ elite individuals are found, they are assigned to *c*_*n*_ crowds according to Euclidean distance. That is, generate a reference point randomly and choose the individual nearest to the reference point as the seed for clustering. Assign the *c*_*s*_* –* 1 individuals nearest to the seed to the same crowd with the seed. Then, remove these individuals from the population and repeat the same process to obtain the rest crowds until all the elite individuals are assigned to the appropriate crowd. After that, the probability models are built in each crowd according to the individuals in each crowd. For the investment amount of endowment policies, the models are Gaussian or Cauchy distribution with expectation equals to the mean value of individuals in the crowd and variance equals to the variance of individuals in the crowd. In terms of the choice of hospitalization policies, the probability models are built based on a histogram method. The probability of hospitalization policy to be chosen equals to the proportion of this choice among the elite individuals in the crowd during this generation. The hospitalization policy appears frequently among the elite individuals in a crowd has a higher probability to be chosen as the hospitalization policy in the next generation.

*Step 3: Generation of new populations in each cluster*. The new population is generated according to the probability models in each crowd. For the investment amount of endowment policies, the new population is sampled by Gaussian or Cauchy distribution according to the performance of these two distributions in the last generation. The distribution appears frequently among the elite individuals in a crowd has a higher probability to be chosen as the distribution for the next generation. In terms of the choice of hospitalization policy, the roulette wheel selection method is applied to determine the decision of hospitalization policies in each crowd for the next generation. The *NP* newly generated individuals and the original *NP* individuals are combined to constitute the candidate population for the next generation. The *NP* better individuals in the candidate population according to dominated rank and crowding distance are chosen to form the population of the next generation.

*Step 4: Termination Check.* While the termination criterion is satisfied, the Pareto-optimal solutions with rank 1 in this generation are exported. Otherwise, a new generation is started from Step 2.

The overall procedure of the proposed NSEDA-C is given in Algorithm 3. 
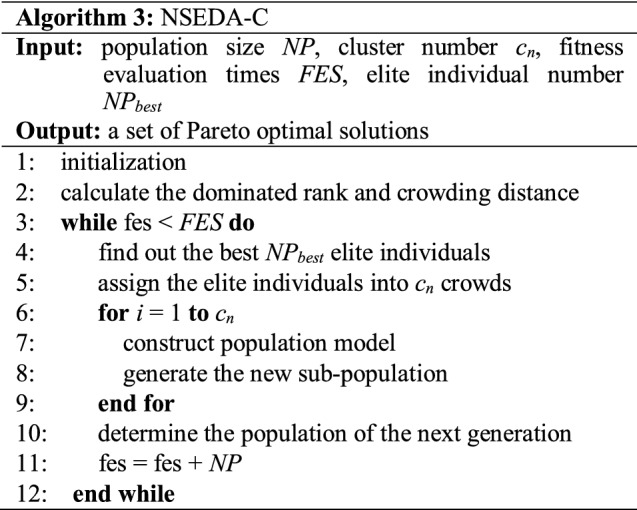


### Simplified simulation approach for the estimation of return

As we have discussed, the group insurance portfolio problem is a scenario-based optimization problem with a high probability that a solution with a higher expectation return will have a higher estimated return in the same simulated scenario. For this feature, a simplified simulation approach is applied for the estimation of return. During the evolution process, the returns for all the individuals are estimated in the same scenario (the illness or death situation of each insured is the same in the simulation of each solution). Instead of the mean value of several simulated results, only one scenario is simulated for the calculation of return in this paper. Due to the scenario-based characteristic and the analysis, it is very likely that the quality of the solutions in terms of the return can be reflected by the return of the solutions in only one scenario. A huge number of fitness evaluations can be saved by the simplified simulation approach.

### Estimation model of the risk

Since the surrogate models have been validated ineffective for the estimation of the risk, a heuristic estimation model is constructed based on the historical data of the insurance policies according to the characteristic of financial optimization problems. The estimation model is calculated by the sum of risk index of endowment policy and hospitalization policy:

9 $$f^{\prime}_{2} ({\varvec{X}},{\varvec{\beta}}) = \sum\limits_{k = 1}^{n} {\left( {\sum\limits_{i = 1}^{{n_{1} }} {\sum\limits_{j = 1}^{{m_{i} }} {x_{ij}^{k} y_{ij} \varpi_{ij}^{k} + r\left( {\beta^{k} } \right)} } } \right)} ,$$

where *n*_1_ is the number of endowment policies and *m*_*i*_ is the number of payment periods of the *i*-th endowment policy. $x_{ij}^{k}$ is the investment amount of the *k*-th insured to the *i*-th endowment policy with the *j*-th payment period. *y*_*ij*_ is the duration of the *j*-th payment period of the *i*-th endowment policy. $\varpi_{ij}^{k}$ is the risk factor of the *i*-th endowment policy with the *j*-th payment period. In addition, *r*(*β*^*k*^) is the risk factor of hospitalization policy for the *k*-th insured. Here, the risk factor $\varpi_{ij}^{k}$ is calculated by

10 $$\varpi_{ij}^{k} = \frac{1}{T}\sum\limits_{t = 0}^{T} {\frac{{\sigma_{ij} (t_{0}^{k} ,t_{0}^{k} + t)}}{{\mu_{ij} (t_{0}^{k} ,t_{0}^{k} + t)}}} ,$$

where *T* is the investment duration considered in the problem and $t_{0}^{k}$ is the purchasement age of the *k*-th insured. *σ*_*ij*_(⋅) and *μ*_*ij*_(⋅) are the standard deviation and mean value calculated from the historical data of the endowment policy.

The risk factor of the hospitalization policy is calculated by

11 $$r(\beta^{k} ) = \sum\limits_{s = 1}^{{n_{2} - \beta^{k} }} {\left( {\left( {1 - \prod\limits_{{t = t_{0}^{k} }}^{{t_{0}^{k} + T}} {\left( {1 - p_{s}^{k} (t)} \right)} } \right) \cdot \overline{z}_{s}^{k} } \right)}$$

where *n*_2_ is the number of hospitalization policies. $p_{s}^{k} (t)$ is the incident rate of the disease in the *s*-th degree for the *k*-th insured at time *t*. $\overline{z}_{s}^{k}$ is the historical average medical expense of the disease in the *s*-th degree for patients with similar condition to the *k*-th insured.

Although the proposed estimation model cannot approximate the accurate value of *f*_2_ (***X***, ***β***), it can estimate the performance of the solutions in terms of the risk. To a large extent, if the value of $f^{\prime}_{2} ({\varvec{X}},{\varvec{\beta}})$ for a solution is larger than another, the value of *f*_2_ (***X***, ***β***) for this solution is very likely to be larger at the same time. The Spearman correlation coefficient [41] is applied to evaluate the accuracy of the rank for the estimated value of the solutions. Spearman correlation coefficient evaluates the correlation between two arrays with the same length based on the degree of agreement in terms of the rank:

12 $$\rho = 1 - \frac{{6\sum\limits_{i = 1}^{N} {d_{i}^{2} } }}{{N(N^{2} - 1)}},$$

where *d*_*i*_ = *x*_*i*_ – *y*_*i*_ is the difference between the ranks of the *i*-th element in the two arrays and *N* is the number of elements in the arrays. *ρ* = 1 implies that the two arrays are sorted exactly the same while *ρ* =  − 1 implies that the two arrays are sorted completely opposite. *ρ* = 0 implies that the two arrays are sorted completely independently. |*ρ|*< 0.4 indicates that the rankings of the two arrays are lowly correlated. 0.4 ≤|*ρ|*< 0.7 indicates that the rankings of the two arrays are moderately related. In addition, 0.7 ≤|*ρ|*< 1 indicates that the rankings of the two arrays are highly correlated.

Experiments are made to validate the rationality of the proposed estimation model. 100 feasible solutions are randomly generated with sample standard deviation calculated in 10,000 scenarios. Their Spearman correlation coefficient of the sample standard deviation with the risk measured by the estimation model is compared with the Spearman correlation coefficient of the sample standard deviation with small amounts of MC simulations (10, 20 and 50) and surrogate models (RBF, BP and GRNN). The experiments are executed 30 times independently to avoid statistical error. The average values of the Spearman correlation coefficients are given in Table 5.

**Table 5:** Average spearman correlation coefficient

	Estimation model	10 *MC*	20 *MC*	50 *MC*	RBF	BP	GRNN
*t*_0_ = 30 45 50, *T* = 20	0.78	0.45	0.71	0.78	− 0.06	0.04	0.02
*t*_0_ = 30 45 50, *T* = 30	0.75	0.49	0.58	0.71	0.09	− 0.01	0.16
*t*_0_ = 30 30 35, *T* = 20	0.82	0.64	0.72	0.84	− 0.02	− 0.08	0.02
*t*_0_ = 30 30 35, *T* = 30	0.77	0.43	0.69	0.74	− 0.22	− 0.05	0.08

It can be discovered that the accuracy of the proposed estimation model is better than 10 and 20 times of MC simulation and comparable with 50 times of MC simulation while a large number of computational resources can be saved. As for the surrogate models, since it has been analyzed that it is inappropriate for scenario-based uncertainty problem, the Spearman correlation coefficients are also very low.

## Experimental results

### Experimental setting

To validate the effectiveness of the proposed approach, experiments are done to compare the NSEDA-C with other algorithms. First, NSEDA with simplified simulation considering the proposed estimation model as the objective of risk is compared with NSEDA considering the mean values and sample standard deviations of small amounts of MC simulation as the objective function value. Secondly, the proposed NSEDA-C is compared with NSEDA without clustering to validate the effectiveness of the clustering approach. Thirdly, the proposed NSEDA-C is compared with other multi-objective algorithms including differential evolution for multi-objective optimization (DEMO) [37], multiple objective particle swarm optimization (MOPSO) [42], DEMO with noise (DEMON) [5] and NSGA-II with *α*-dominance operator (NSGA-II-*α*) [6]. Lastly, NSEDA-C is compared with other two robust optimization algorithms, multi-objective evolutionary algorithm with robust approach (MOEA-R) [43] and reduced Pareto set genetic algorithm with robustness (RPSGA-R) [44].

All the algorithms are executed 30 times independently to reduce the statistical error. The fitness evaluation times for all the algorithms are set as 3 × 10^5^ for fairness. The population size of NSEDA-C is set as 1000 [14]. The population size of NSEDA considering 10, 20 and 50 times of MC simulation are set as 100, 50 and 20, respectively. The population size of DEMO and DEMON are set as 50 [5, 37]. The population size and archive size of MOPSO are set as 40 and 200, respectively [42]. The population size of NSGA-II-*α* is set as 100 [6]. The cluster number of NSEDA-C is set as 10 and the population size of elite individuals of NSEDA-C is set as 450 [14]. The mutation rate and crossover rate of NSGA-II-*α* are set as 0.02 and 0.9, respectively [6]. The crossover rate in DEMO and DEMON is set as 0.9 [5, 37]. The reduction rate in MOEA-R is set as 0.5 [43]. The dispersion parameter in RPSGA-R is set as 0.5 [44].

In the experiments, several real-life insurance products are considered in the following four situations, where *t*_0_ is the initial ages of different insureds in the group and *T* is the time duration considered during investment:

1. *t*_0_ = 30 45 50, *T* = 20;
2. *t*_0_ = 30 45 50, *T* = 30;
3. *t*_0_ = 30 30 35, *T* = 20;
4. *t*_0_ = 30 30 35, *T* = 30.

All the solutions obtained from the above algorithms are considered together and the nondominated solutions among them are applied to construct the approximated Pareto Front of the group insurance optimization problem which is denoted as *P*. The approximated Pareto Front obtained by each algorithm is denoted as *A*. The inverted generational distance (*IGD*) and spacing (*S*) are employed to evaluate the performance of the algorithms [45]:

13 $$IGD(A,P) = \frac{{\sum_{i = 1}^{\left| P \right|} {d(p_{i} ,A)} }}{\left| P \right|},$$

where *d*(*p*_*i*_, *A*) is the minimum Euclidean distance from the *i*-th member in *P* to the members in *A*. A small *IGD* value implies that *A* is close to *P* indicating a good performance of the algorithm:

14 $$S(A) = \sqrt {\frac{1}{\left| A \right| - 1}\sum\limits_{i = 1}^{\left| A \right|} {(\overline{d} - d_{i} )^{2} } } ,$$

15 $$d_{i} = \min \left( {\left| {f_{1i} - f_{1j} } \right| + \left| {f_{2i} - f_{2j} } \right| } \right),$$

where $\overline{d}$ is the mean value of *d*_*i*_ and *f*_*ij*_ is the *i*-th objective value of the *j*-th solution in *A*. A small *S* value implies that the members in *A* are spaced almost equidistantly indicating a good performance of the algorithm.

### The estimated Pareto Front of each algorithm

**Fig. 3:**
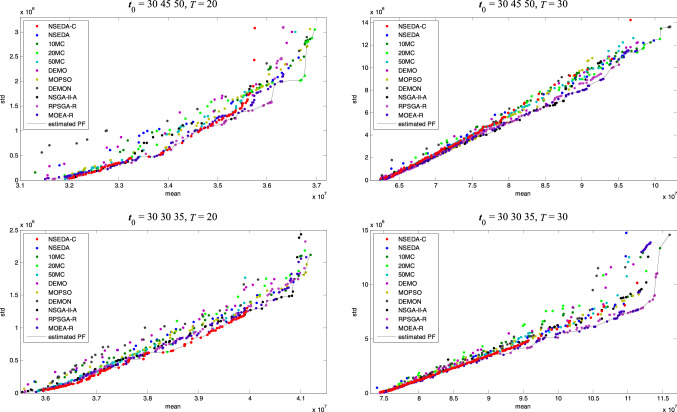
The estimated Pareto Fronts of each algorithm

For an intuitive comparison of algorithms, the estimated Pareto Fronts optimized by each algorithm are printed as points with different colors in Fig. 3. The overall estimated Pareto Fronts gained by all the algorithms are indicated by black lines in Fig. 3. The mean value and sample standard deviation of the solutions are re-evaluated in 10,000 scenarios to obtain a more accurate objective value. The estimated Pareto Fronts in Fig. 3 are constructed by the nondominated solutions in terms of the new objective value. It can be discovered that compared with other algorithms, the estimated Pareto Fronts gained by the proposed NSEDA-C (red points) are closer to the overall estimated Pareto Fronts in all of the four situations. That is to say, the performance of NSEDA-C is better than other algorithms on the whole. However, further calculations of the indicators are needed to confirm this cognition. Since we do not know the true Pareto Fronts of the group insurance portfolio problem in advance, the overall estimated Pareto Fronts gained by all the algorithms are applied to evaluate the performances of the algorithms.

#### Comparison results

The average *IGD* and *S* value in 30 independent runs for all of the algorithms are given in Tables 6 and 7. To verify the significance of the difference, the *p* values of *IGD* for Wilcoxon rank sum test [46] compared with the proposed NSEDA-C are also given in Tables 6 and 7. The p-values smaller than 0.05 indicate that the difference are significance and are highlighted with bold. What's more, the algorithms with the smallest IGD and S value are also highlighted with bold to indicate their good performances.

**Table 6:** Average *IGD* and *S* value for all the algorithms

		NSEDA-C	NSEDA	10 *MC*	20 *MC*	50 *MC*	DEMO	MOPSO	DEMON	NSGA-II-*α*
***t***_**0**_** = 30 45 50** ***T* = 20**	***IGD***	**5.781E + 04**	7.105E + 04	8.296E + 04	9.298E + 04	1.073E + 05	1.007E + 05	9.622E + 04	3.954E + 07	8.739E + 04
	***p* value**	–	**3.020E-11**^**+**^	**3.020E-11**^**+**^	**3.020E-11**^**+**^	**4.573E-09**^**+**^	**5.494E-11**^**+**^	**3.020E-11**^**+**^	**3.020E-11**^**+**^	**3.020E-11**^**+**^
	***S***	8.361E + 05	1.490E + 06	1.209E + 06	2.092E + 06	1.045E + 06	1.688E + 06	**4.884E + 05**	8.935E + 06	7.854E + 05
***t***_**0**_** = 30 45 50** ***T* = 30**	***IGD***	**2.853E + 07**	2.854E + 07	2.857E + 07	2.859E + 07	2.857E + 07	2.857E + 07	2.855E + 07	7.305E + 07	2.855E + 07
	***p* value**	–	**1.777E-10**^**+**^	**4.077E-11**^**+**^	**6.696E-11**^**+**^	**3.474E-10**^**+**^	**5.072E-10**^**+**^	**2.034E-09**^**+**^	**4.975E-11**^**+**^	**3.835E-06**^**+**^
	***S***	3.205E + 06	3.723E + 06	5.789E + 06	5.912E + 06	4.306E + 06	4.749E + 06	1.759E + 06	2.968E + 07	**1.575E + 06**
***t***_**0**_** = 30 30 35** ***T* = 20**	***IGD***	**2.838E + 04**	4.403E + 04	5.218E + 04	5.208E + 04	7.930E + 04	5.229E + 04	5.517E + 04	5.438E + 04	4.966E + 04
	***p *value**	–	**3.020E-11**^**+**^	**3.020E-11**^**+**^	**3.020E-11**^**+**^	**3.020E-11**^**+**^	**3.020E-11**^**+**^	**3.020E-11**^**+**^	**3.020E-11**^**+**^	**3.020E-11**^**+**^
	***S***	**2.716E + 05**	3.795E + 05	7.013E + 05	9.552E + 05	1.104E + 06	5.388E + 05	6.588E + 05	6.284E + 06	3.720E + 05
***t***_**0**_** = 30 30 35** ***T* = 30**	***IGD***	**1.109E + 07**	1.109E + 07	1.111E + 07	1.112E + 07	1.113E + 07	1.111E + 07	1.112E + 07	9.839E + 07	1.111E + 07
	***p* value**	–	**3.020E-11**^**+**^	**3.020E-11**^**+**^	**3.020E-11**^**+**^	**7.288E-03**^**+**^	**3.020E-11**^**+**^	**3.020E-11**^**+**^	**6.121E-10**^**+**^	**3.020E-11**^**+**^
	***S***	**2.357E + 06**	2.445E + 06	4.693E + 06	5.854E + 06	4.193E + 06	4.628E + 06	4.161E + 06	2.269E + 07	4.093E + 06

The p-values smaller than 0.05 indicate that the difference are significance and are highlighted with bold. What's more, the algorithms with the smallest IGD and S value are also highlighted with bold to indicate their good performances

**Table 7:** Average *IGD* and *S* value for NSEDA-C and robust optimization algorithms

		NSEDA-C	MOEA-R	RPSGA-G
***t***_**0**_** = 30 45 50** ***T* = 20**	***IGD***	**5.781E + 04**	8.100E + 04	7.862E + 04
	***p* value**	**–**	**2.922E-09**^**+**^	**5.072E-10**^**+**^
	***S***	8.361E + 05	4.091E + 05	**2.518E + 05**
***t***_**0**_** = 30 45 50** ***T* = 30**	***IGD***	**2.853E + 07**	2.854E + 07	2.855E + 07
	***p* value**	**–**	**1.868E-05**^**+**^	**6.528E-08**^**+**^
	***S***	3.205E + 06	**7.029E + 05**	8.204E + 05
***t***_**0**_** = 30 30 35** ***T* = 20**	***IGD***	**2.838E + 04**	4.770E + 04	4.798E + 04
	***p* value**	**–**	**3.020E-11**^**+**^	**3.020E-11**^**+**^
	***S***	2.716E + 05	2.245E + 05	**1.368E + 05**
***t***_**0**_** = 30 30 35** **T = 30**	***IGD***	**1.109E + 07**	1.110E + 07	1.110E + 07
	***p* value**	**–**	**6.722E-10**^**+**^	**9.919E-11**^**+**^
	***S***	2.357E + 06	1.116E + 06	**9.964E + 05**

The p-values smaller than 0.05 indicate that the difference are significance and are highlighted with bold. What's more, the algorithms with the smallest IGD and S value are also highlighted with bold to indicate their good performances.

#### Comparison among NSEDA and small amount of MC

To verify the effectiveness of the proposed simplified simulation strategy and the estimation model, NSEDA with these two approaches are compared with NSEDA considering small amounts of MC simulation as objective function values. Since the simplified simulation strategy only generated one scenario for the evaluation of the return, it has to be integrated with the estimation model, otherwise the risk cannot be evaluated.

It can be discovered from Table 6 that the NSEDA with the proposed approaches outperforms the NSEDA considering small amounts of MC simulation. Specifically, in the first situation, the average *IGD* for NSEDA is smaller than the average *IGD* for the other three algorithms significantly. As for the average *S* value, it is a bit larger than the algorithms considering 10 simulations and 50 simulations. On one hand, the difference in *S* value is much smaller than the difference in *IGD* considering the magnitude. On the other hand, the value of *IGD* is much more significant than the value of *S* for the evaluation of the multi-objective algorithms considered in this paper. Therefore, NSEDA with the proposed approaches outperforms the other three algorithms in general in the first situation. In the second and fourth situations, average *IGD* values for NSEDA are smaller than the average *IGD* values for the other three algorithms slightly. As for the average *S* values, they are also smaller than the other algorithms. Therefore, it can be concluded that NSEDA with the proposed approaches outperforms the other three algorithms in the second and fourth situations. In the third situation, both the average *IGD* value and average *S* value for NSEDA are smaller than the other algorithms significantly. Therefore, it can easily draw a conclusion that NSEDA with the proposed approaches outperforms the other three algorithms in the third situation. Therefore, the effectiveness of the proposed simplified simulation strategy and the estimation model has been validated.

#### Comparison between NSEDA-C and NSEDA

To verify the effectiveness of the clustering method, NSEDA-C with clustering is compared with NSEDA without clustering. It can be discovered that in all the situations, both the average *IGD* value and average *S* value for NSEDA-C are smaller than NSEDA. Therefore, it can easily draw a conclusion that NSEDA-C outperforms NSEDA.

The effectiveness of the clustering method has been validated. This is because the group insurance portfolio optimization problem considered in this paper is a multimodal problem with several peaks. With the help of clustering method, more peaks can be discovered during the evolution process which leads to a more promising performance of the algorithm.

#### Comparison among NSEDA-C and other multi-objective algorithms

To verify that the NSEDA-C is effective for the proposed group insurance portfolio problem, NSEDA-C is compared with other multi-objective algorithms. Four multi-objective algorithms including two deterministic algorithms (DEMO and MOPSO) and two uncertain algorithms (DEMON and NSGA-II-*α*) are chosen as comparison algorithms.

In all the situations, the average *IGD* value for NSEDA-C is smaller than the average *IGD* values for the other four algorithms significantly. As for the average *S* value, it is a bit larger than MOPSO and NSGA-II-*α* in the first and second situations. Since the *IGD* value is more important for the evaluation of the algorithms in the multi-objective problem considered in this paper, the performance of NSEDA-C is regarded as the best among five algorithms. The effectiveness of the NSEDA-C can be validated through the experimental results.

#### Comparison among NSEDA-C and robust optimization algorithms

To make the results more convincing, we further compared the proposed NSEDA-C with two robust optimization algorithms, MOEA-R and RPSGA-R.

The comparison results are given in Table 7. It can be discovered that the average *IGD* value for NSEDA-C is significantly smaller than MOEA-R and RPSGA-R in all the situations. Since there are robust handling approach embedding in MOEA-R and RPSGA-R, the average *S* value for NSEDA-C is larger than the other two algorithms. However, the value of *IGD* is much more important for the evaluation of the algorithms in the multi-objective algorithms considered in this paper. Therefore, the proposed NSEDA-C is more suitable to deal with the group insurance portfolio problem compared with these two robust optimization algorithms.

## Conclusions

In this paper, a nondominated sorting estimation of distribution algorithm with clustering is proposed to deal with the group insurance portfolio problem with two objectives, return maximization and risk minimization. To improve the effectiveness of the algorithm, a simplified simulation approach is applied to estimate the return taking advantage of the scenario-based characteristic of the problem. Moreover, since the surrogate models are unsuitable for the scenario-based problem, a heuristic estimation model is designed to estimate the risk of the problem.

This paper explores the application of the simplified simulation approach and the problem-based heuristic estimation model to scenario-based uncertain multi-objective optimization problems. However, some procedures of insurance investment have been ignored when considering group insurance portfolio problem in this paper, such as surrender and additional investment, which can be added in the future work.
